# Comparative Pharmacokinetics and Allometric Scaling of Carboplatin in Different Avian Species

**DOI:** 10.1371/journal.pone.0134177

**Published:** 2015-07-29

**Authors:** Gunther Antonissen, Mathias Devreese, Siegrid De Baere, Tom Hellebuyck, Isabel Van de Maele, Lieze Rouffaer, Hendrickus J. J. Stemkens, Patrick De Backer, An Martel, Siska Croubels

**Affiliations:** 1 Department of Pharmacology, Toxicology and Biochemistry, Faculty of Veterinary Medicine, Ghent University, Merelbeke, Belgium; 2 Department of Pathology, Bacteriology and Avian Diseases, Faculty of Veterinary Medicine, Ghent University, Merelbeke, Belgium; 3 Department of Small Animal Medicine and Clinical Biology, Faculty of Veterinary Medicine, Ghent University, Merelbeke, Belgium; NIH, UNITED STATES

## Abstract

The use of chemotherapeutics as a possible treatment strategy in avian oncology is steadily increasing over the last years. Despite this, literature reports regarding dosing strategies and pharmacokinetic behaviour of chemotherapeutics in avian species are lacking. The aim of the present study was to investigate the pharmacokinetics of carboplatin in a representative species of the order of *Galliformes*, *Anseriformes*, *Columbiformes* and *Psittaciformes*. Eight chickens, ducks and pigeons and twenty-eight parakeets were administered carboplatin intravenously (5 mg/kg body weight). A specific and sensitive liquid chromatography-tandem mass spectrometry method was developed and validated for quantification of the free carboplatin in plasma of the four birds species (limit of quantification: 20 ng/mL for chicken and duck, 50 ng/mL for pigeon and 100 ng/mL for parakeets). Non-compartmental pharmacokinetic analysis and allometric scaling demonstrated a significant correlation (R^²^ = 0.9769) between body weight (BW) and elimination half-life (T_1/2el_). T_1/2el_ ranged from 0.41 h in parakeets (BW: 61 ± 8 g) to 1.16 h chickens (BW: 1909 ± 619 g). T_1/2el_ is a good parameter for dose optimization of carboplatin in other avian species, since also the previously reported T_1/2el_ in cockatoos (average BW: 769 ± 68 g) of 1.00 h corresponds to the results obtained in the present study.

## Introduction

Neoplastic diseases are frequently reported in commonly kept pet birds of the order of *Galliformes*, *Anseriformes*, *Columbiformes* and *Psittaciformes* [1–5]. The use of chemotherapeutics as a possible treatment strategy in avian oncology is steadily increasing over the last years [6]. Defining the optimal treatment protocol for tumours in birds, however is still largely empirical. Treatment protocols used in birds have been extrapolated from other companion animals such as dogs and cats or humans [7]. As uro-genital and integumentary neoplastic processes are amongst the most common tumours in birds [1, 2, 5], the use of platinum-based antineoplastic agents is of great interest. Carboplatin [cis-diammine (1,1-cyclobutanedicarboxylato) platinum (II)] (CPT) is a water-soluble second generation platinum compound used to treat ovarian and cervix carcinomas and bladder cancers in humans. Carboplatin was developed to reduce adverse effects such as myelosuppression, nephrotoxicity and nausea associated with the use of cisplatin, a first-generation platinum agent [8]. Cisplatin and CPT form reactive intracellular platinum complexes that bind to nucleophilic groups in DNA, producing both inter- and intra-strand crosslinks that inhibit DNA replication, RNA transcription, and protein synthesis, resulting in apoptosis [9]. In birds, several cases have been reported in which CPT was applied to treat neoplastic disorders with variable outcome. Intraosseus (IO) administration of CPT (3 doses of 5 mg/kg BW, at 4 week intervals) showed to be successful to treat pancreatic duct adenocarcinoma in a Green-winged macaw (*Ara chloroptera*) [10]. Furthermore, intravenous (IV) CPT administration (4 doses of 8.5–15 mg/kg BW, at 4–5 weeks intervals) in a duck (*Anas platyrhynchos*) reduced the size of a Sertoli cell tumour by 25%, and improved the duck’s clinical condition for 12 months [11]. IV administration of CPT (3 doses of 5 mg/kg, at 4 week intervals) in a budgerigar (*Melopsittacus undulatus*) with a renal adenocarcinoma initially improved the clinical condition of the bird. Nevertheless, after three months, the bird’s condition gradually deteriorated and the mass was again enlarged [12].

Despite the clinical application of CPT in different avian species, a suitable treatment protocol, based on pharmacokinetic (PK) data, is lacking. Only one study described the PK behaviour of CPT in sulfur-crested cockatoos [13]. Since the myelosuppressive toxicity of CPT is dose-dependent, and determined by the area under the plasma concentration-time curve (AUC), there is an urgent need for therapeutic drug monitoring and data regarding the PK characteristics of CPT in birds [13, 14]. Similar to other drugs such as non-steroidal anti-inflammatory drugs (NSAIDs) and antibiotics, the PK can be significantly influenced by the biological specifics of the avian species and order, therefore species-specific PK studies in birds are needed [15, 16].

Therefore the aims of present study were first, to develop and validate a sensitive and specific liquid chromatography-tandem mass spectrometric (LC-MS/MS) method for quantification of CPT in avian plasma. Secondly, to study the PK of CPT in a representative species of four different avian orders, namely *Galliformes*, *Anseriformes*, *Colombiformes* and *Psittaciformes*. Thirdly, to apply allometric scaling to evaluate whether the PK results can be extrapolated to other avian species (e.g. cockatoos) and eventually to other animal species and humans.

## Materials and Methods

### Chemicals, products and reagents

CPT used for the birds (10 mg/mL water) was obtained from Hospira (Brussels, Belgium) and the analytical standard of CPT used for the analytical experiments was obtained from Sigma-Aldrich (Bornem, Belgium) and stored at 4°C. Water and acetonitrile (ACN) were of LC–MS grade and obtained from Biosolve (Valkenswaard, the Netherlands). Glacial acetic acid was of analytical grade and obtained from VWR (Leuven, Belgium).

### Animal experiments

The PK studies were carried out in chickens (ISA Brown layer, *Gallus gallus*), ducks (Call Duck, *Anas platyrhynchos*), pigeons (racing pigeon, *Columbia livia* forma domestica) and parakeets (English budgerigar, *Melopsittacus undulatus*). CPT was administered to eight (four male / four female) chickens, ducks and pigeons with an average ± SD body weight (BW) of 1909 ± 619 g, 756 ± 101 g and 470 ± 67 g, respectively, and twenty-eight (fourteen male / fourteen female) parakeets with an average BW of 61 ± 8 g. All birds were clinically healthy and on average one year old (range 9–13 months). Throughout one week acclimatisation period, animals were housed in group per species, and had *ad libitum* access to feed and drinking water.

Subsequently, eight hours before the start of the experiment, the animals were deprived of feed. Birds were anaesthetised for 10 min with isoflurane (IsoFlo, Abbott Animal Health, Waver, Belgium) and oxygen inhalation. Body temperature during anaesthesia was maintained using a heat mat. CPT was administered IV in the *vena cutanea ulnaris superficialis* (wing vein) at a dose of 5 mg/kg BW. This was performed by cannulating the vein in chickens, ducks and pigeons with a 26-gauge IV catheter (Terumo versatus winged IV catheter 26G 0.64x19 mm, Terumo Europe, Leuven, Belgium). In parakeets, IV administration was performed via a 27-gauge cannula scalp vein set (Venoflux, 0.40x16 mm, tube 7 cm, Vygon, Brussels, Belgium). CPT (Hospira, Brussels, Belgium) was diluted in 5% dextrose solution (5 mg CPT/mL for chickens, ducks and pigeons, and 2.5 mg CPT/mL for parakeets) and was infused in the vein over 3 min. After CPT administration the catheter or cannula was flushed by an equal volume of 5% dextrose solution. Subsequently, the IV catheter or cannula was removed and anaesthesia was terminated 6 min after the start of the carboplatin infusion.

Blood samples (0.5 mL in chickens and ducks and 0.2 mL in pigeons and parakeets) were collected in heparinised tubes from the *vena metatarsalis plantaris superficialis* (chickens, ducks and pigeons) or the *vena jugularis* (parakeets) before administration (t = 0) and at 3 min (end of infusion), 5, 10, 20, 30, 45 min and 1, 2, 3, 4, 6, 8, 12 and 24 h after the start of the infusion. The blood samples before administration and at 3 and 5 min after the start of the infusion were taken during anaesthesia. In chickens, ducks and pigeons, blood was drawn at each sampling point from each animal. For parakeets, a sparse sampling protocol was applied because of the limited volume of blood that can be drawn from these birds. Therefore, all sampling points were randomly allocated to different birds, with three sampling points per bird. Blood samples were centrifuged (2851 x *g*, 10 min, 4°C) and plasma was stored at ≤ -70°C until analysis.

### Ethics Statement

The animal experiment was approved by the Ethical Committee of the Faculty of Veterinary Medicine and Bioscience Engineering of Ghent University (EC 2012/109).

### Quantification of carboplatin in plasma

To 50–100 μL of plasma (depending on the bird species) were added 50–100 μL of water followed by a vortex mixing and filtration step (Amicon Ultra 0.5 mL centrifugal filter, MWCO 30 kDa, Millipore, Brussels, Belgium). Next, samples were centrifuged (8751 x *g*, 30 min, 4°C) and the filtrate was transferred to an autosampler vial before injection onto the LC-MS/MS instrument.

The chromatographic system consisted of an Alliance type 2695 HPLC separations module with column heater and cooling device (Waters, Milford, USA). Chromatographic separation was achieved using a Hypersil Gold aQ C18 column (150 x 4.6 mm i.d., d.p.: 3.0 μm), in combination with a guard column of the same type, from ThermoFisher Scientific (Breda, The Netherlands). Mobile phase A was 0.1% acetic acid in water, while mobile phase B was ACN. A gradient elution program was performed: 0–4 min: 99% A/1% B, 4–6 min: linear to 30% A, 6–7 min: 30% A/70% B, 7–8 min: linear to 99% A, 8–12 min: 99% A/1% B. The flow rate was set at 0.7 mL/min.

The LC column effluent was pumped to a Quattro Ultima triple quadrupole mass spectrometer (Waters, Altrincham, UK), equipped with an electrospray ionization (ESI) ion source, operated in the positive ion mode. A splitter device was used to direct 50% of the LC effluent to the mass spectrometer and 50% to the waste. The instrument was tuned by direct infusion of a 1 μg CPT/mL working solution. The following parameters were retained for optimal CPT detection: capillary voltage: 3 kV, cone voltage: 45 V, source temperature: 120°C, desolvation temperature: 250°C, cone gas flow: 55 L/h, desolvation gas flow: 845 L/h.

Acquisition was performed in the selected reaction monitoring (SRM) mode. Following SRM transitions were monitored and used for identification and quantification of CPT: *m/z* 372.0 > 229.0 and 372.0 > 293.9 (collision energy: 17 eV), respectively. Quantification was performed with the MassLynx software v4.0 (Micromass), using the above mentioned product ions.

The method was validated for plasma from the four different avian species according to a validation protocol previously described by [17]. A set of parameters that were in compliance with the recommendations and guidelines defined by the European Community and with criteria described in the literature, were evaluated [18–20]. These parameters encompassed linearity (correlation coefficient, r, and goodness-of-fit coefficient, g), within- and between-run accuracy and precision, limit of quantification (LOQ) and carry-over.

### Pharmacokinetic analysis and allometric scaling

Non-compartmental PK analysis was performed using WinNonlin version 6.3 (Pharsight, St-Louis, MI, USA). Following main PK parameters after IV administration were calculated for the different avian species: area under the plasma concentration-time curve from time 0 to the last sample point with values above the LOQ (AUC_0-t_), area under the plasma concentration-time curve from time 0 to infinite (AUC_0-inf_), plasma concentration at time zero (C_0_), elimination rate constant (k_e_), elimination half-life (T_1/2el_), volume of distribution (Vd) and total body clearance (Cl). Allometric scaling was applied on T_1/2el_, Vd and Cl based on the following power function [21]: *Y = a*.*W* ^*b*^, where *Y* is the value of the respective PK parameter, *a* is the coefficient of the intercept of the trend line, *W* is the body weight and *b* is the slope of the trend line.

### Statistical analysis

All PK parameters were compared between avian species and between genders within one species with one-way ANOVA and a Student’s *t*-test, respectively (SPSS 21.0, IBM, USA). The level of significance was set at 0.05.

## Results

### LC-MS/MS method

Due to the broad concentration range of CPT in plasma (between 20 and 50 000 ng/mL), matrix-matched calibration graphs (1/x weighed) were quadratic and split in a low and high concentration range. The method of external standardization was applied, since the analyte (oxaliplatin) that was used for internal standardization during preliminary experiments had a limited stability in extracted samples in contrast to CPT. As a consequence, the quantification of CPT in plasma was impaired by applying the method of internal standardization. Good correlation between analyte concentrations and detected responses was demonstrated for all matrices and both concentration ranges, with r values ranging between 0.9985 and 0.9999 and g values between 3.43 and 7.29% (S1 Table). The within- and between-run accuracy and precision were evaluated at minimally 3 concentration levels and fell within the acceptability ranges (S2 Table). The LOQs were 20 ng/mL for chicken and duck, 50 ng/mL for pigeon and 100 ng/mL for parakeets (S1 Table). No carry-over was present as there was no CPT detected in the solvent sample injected after the highest calibrator.

### Plasma pharmacokinetic analysis and allometric scaling

All birds tolerated the anaesthetic procedure as well as the CPT infusion, and recovered without complications. During the animal experiment, no clinical signs of CPT toxicity were observed in any of the birds. All birds were alert, had a normal feed intake and normal droppings, and no vomiting was observed.

**Fig 1:**
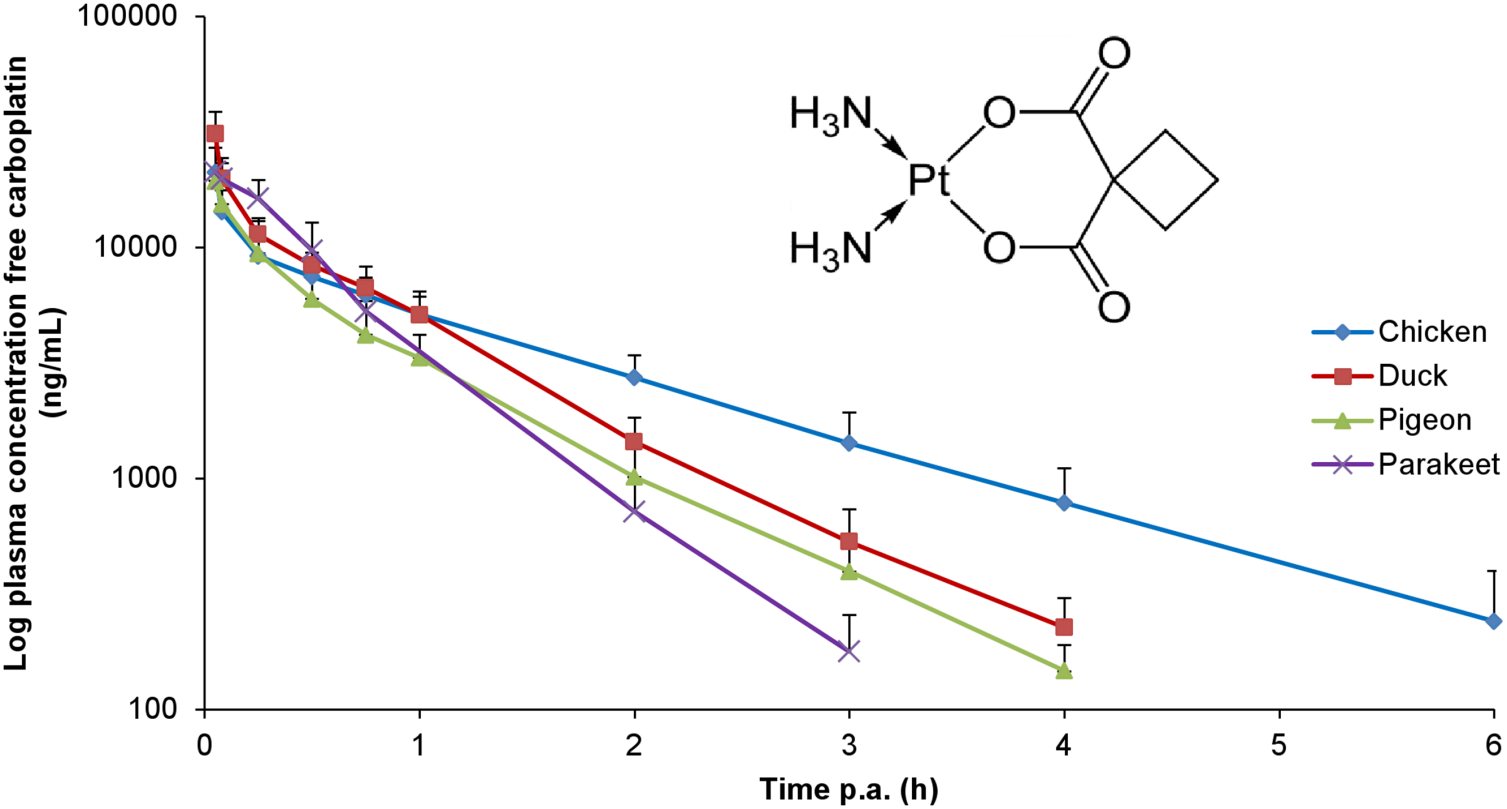
Plasma concentration-time profiles of free carboplatin in different avian species (chicken, duck, pigeon and parakeet) after intravenous administration of 5 mg carboplatin/kg body weight. Values are presented as mean + SD. The insert displays the chemical structure of carboplatin.

**Fig 2:**
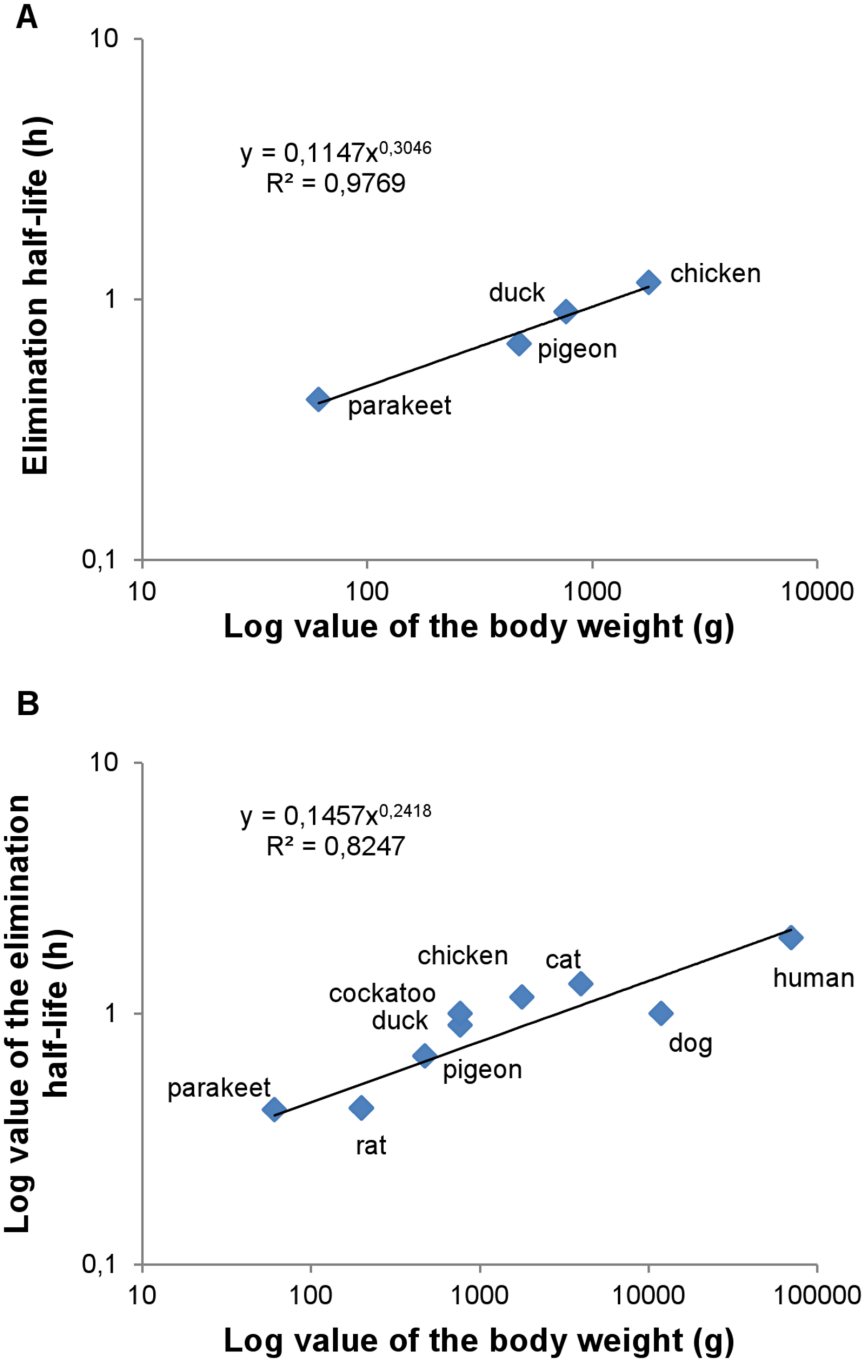
Allometric scaling of the elimination half-life of free carboplatin in different avian species derived from the present study (a) and in all species for which literature data are available (b). Elimination half-life values of rat, cockatoo, cat, dog and human were derived from [13, 22–25].

The plasma concentration-time profile and the main PK characteristics after IV administration of CPT to the different avian species are presented in Fig 1 and Table 1, respectively. There were no significant gender differences within one species (results not shown). Between avian species, significant differences were detected for AUC_0-t,_ AUC_0-inf,_ k_e_ and T_1/2el_, but not for Vd and Cl. Allometric scaling revealed a good correlation between T_1/2el_ and BW of the bird species (R² > 0.97), as depicted in Fig 2a. Furthermore, allometric scaling of the T_1/2el_ was performed in different avian species derived from this study and in all species for which literature data are available, namely rat [22], cockatoo [13], cat [23], dog [24] and human [25]. Results are presented in Fig 2b. A moderate correlation between T_1/2el_ and BW of the different species (R² > 0.82) was demonstrated.

**Table 1:** Main pharmacokinetic parameters of free carboplatin after intravenous administration (5 mg/kg BW) to chickens, ducks, pigeons and parakeets.

	Chicken	Duck	Pigeon	Parakeet
AUC_0-t_ (h.μg/mL)	17.43 ± 3.39 ^a^	16.68 ± 2.79 ^a,b^	11.21 ± 2.81 ^b^	14.20 ± 1.73 ^a,b^
AUC_0-inf_ (h.μg/mL)	17.87 ± 3.52 ^a^	16.76 ± 2.81 ^a,b^	11.36 ± 2.78 ^b^	14.31 ± 1.72 ^a,b^
C_0_ (μg/mL)	41.27 ± 12.59 ^a^	69.67 ± 24.55 ^a^	32.91 ± 22.26 ^a^	52.69 ± 40.59 ^a^
k_e_ (1/h)	0.63 ± 0.13 ^a^	0.80 ± 0.14 ^a,b^	1.04 ± 0.11 ^b^	1.71 ± 0.22 ^c^
T_1/2el_ (h)	1.16 ± 0.22 ^a^	0.90 ± 0.15 ^a,b^	0.68 ± 0.07 ^b^	0.41 ± 0.05 ^c^
Vd (mL/kg)	494.82 ± 124.18 ^a^	398.25 ± 72.75 ^a^	517.45 ± 188.25 ^a^	213.32 ± 35.39 ^a^
Cl (mL/h/kg)	293.24 ± 62.17 ^a^	309.82 ± 53.02 ^a^	520.59 ± 179.94 ^a^	355.44 ± 41.87 ^a^

Values are presented as mean ± SD.

AUC_0-t_: area under the plasma concentration-time curve from time 0 to the last sample point with values above the limit of quantification, AUC_0-inf_: area under the plasma concentration-time curve from time 0 to infinite, C_0_: plasma concentration at time 0, k_e_: elimination rate constant, T_1/2el_: elimination half-life, Vd: volume of distribution, Cl: total body clearance.

Values within one parameter with a different superscript are statistically different at p < 0.05.

## Discussion

### LC-MS/MS method

Several methods for detection of free CPT in biological fluids have been reported in literature. In the last decade, LC-MS/MS became the method of choice due to its high sensitivity and selectivity. It has been reported that sample treatment consisting of solid-phase extraction (SPE), e.g. using C_18_, C_2_, CN and NH_2_ cartridges, generally leads to limited extraction recoveries [26]. CPT is a strong hydrophilic compound (log P = -2.3, chemical structure is shown in Fig 1) [27] and cannot be retained on most reverse-phase SPE stationary phases, thus compromising the sensitivity and reproducibility of the method. In contrast, strong cation exchange (SCX) columns contain benzenesulfonic acid anions which interact with the amine groups on carboplatin, resulting in an adequate retention of CPT [28]. Nevertheless, these cartridges seem to be very sensitive to washing steps and elution volumes inducing variable extraction results [28]. On- and off-line protein precipitation has been described as sample pretreatment procedure for human plasma, although significant matrix effect (signal suppression) was present yielding low analyte responses. The matrix effect was overcome by combining the precipitation of proteins with phospholipid removal (HybridSPE-PPT). However only low extraction recoveries were obtained (range 0.1–32.4% depending on the solvent) [26]. For PK studies where a large number of samples need to be analyzed, high-throughput sample preparation is crucial. In the present study it was opted to dilute the sample by a factor 2, followed by the physical removal of proteins and molecules with a molecular mass above 30 kDa by ultrafiltration. This ultrafiltration is crucial as a fraction of the drug is unavailable because of covalent binding to plasma proteins (accounting for 40% of the total CPT in humans [29]). Therefore, only the free (unbound) fraction of CPT has to be quantified [30]. Chromatographic separation of CPT is commonly performed by a combination of an organic solvent (ACN or methanol) and water with a volatile acid (e.g. acetic acid or formic acid) [8]. The most optimal chromatographic conditions were obtained by a combination of 0.1% acetic acid in water and ACN. A Hypersil Gold aQ column was used as stationary phase for optimal retention of this polar analyte. Concerning the MS/MS parameters, the highest sensitivity was obtained for the protonated parent compound measured in the positive ESI mode and the followed SRM traces were in accordance with literature [26, 31].

### Pharmacokinetic analysis and allometric scaling

The dose used in this PK study (5 mg/kg BW) was based on a previous study in cockatoos [13]. Dosing of antineoplastic drugs should be done very carefully because of their narrow therapeutic-toxic range. Chemotherapeutics are commonly dosed based on the body surface area (BSA) of a patient instead of the BW, because the BSA is considered to be a better indicator of the metabolic mass. However, limited information is available to determine the BSA in exotic animals such as pet birds [32]. Therefore, a major goal of this study was to determine whether dose extrapolation between different avian species can be performed based on the correlation of one or more PK characteristics and the BW. Attempts have previously been made to allometrically scale the T_1/2el_ with BW in birds for NSAIDs and antibiotics, however, with marginal success. Reasons for this lack of correlation are still poorly understood but are thought to be related to species dependent elimination processes and differences in protein binding [16]. In our study, a clear correlation (R² > 0.97) was demonstrated between BW of the bird species and T_1/2el_ as depicted in Fig 2a and T_1/2el_ ranged between 0.41 h for parakeets (average BW: 61 g) and 1.16 h for chickens (average BW: 1909 g) (Table 1). This indicates that, based on the BW, the T_1/2el_ can be estimated in avian species. Indeed, the T_1/2el_ of ducks (average BW: 756 g), namely 0.90 h (Table 1), corresponds well with the previously described T_1/2el_ in cockatoos (average BW: 769 ± 68 g) of 1.00 h [13]. Furthermore, Siddik et al. [22], Bailey et al. [23], Gaver et al. [24] and van der Vijgh [25] demonstrated a T_1/2el_ of CPT in rats, cats, beagle dogs and humans of 0.41, 1.31, 1 and 2 h, respectively [22–25], which could be scaled with an acceptable correlation (R² = 0.83) as well (Fig 2b). However, it should be taken into account the use of different PK models can lead to slight differences in T_1/2el_ as well. CPT in humans displays a three-compartmental course, with a distribution (T_1/2α_ = 0.38 h), initial (T_1/2β_ = 2 h) and terminal (T_1/2γ_ = 5.8 days) elimination half-life [25]. Siddik et al. [22] applied a two-compartmental model for CPT in rats (T_1/2α_ = 0.036 h, T_1/2β_ = 0.41 h), whereas in the present study in birds and the other described studies in cockatoos [13], cats [23] and dogs [24] a non-compartmental model was used [13, 23, 24]. The values of T_1/2el_ obtained from the non-compartmental designs resemble the T_1/2β_ from the two- or three-compartmental model as they are calculated based on the last sampling points.

The results regarding T_1/2el_ indicate that allometric scaling can most probably be applied within one class, but it is not always applicable between classes. In accordance to the obtained results in avian species, [33] allometrically scaled the PK characteristic of 44 drugs across veterinary and laboratory animal species and concluded that T_1/2el_ is the most robust parameter for interspecies scaling. In their study, the allometric exponent (*b*) averaged around 0.24 ± 0.09 with 65% of the *b*’s between 0.19 and 0.32. Indeed, the value most cited in literature in general for this term is 0.25 [34]. These values correspond well with the value for *b* obtained in this study, namely 0.30 within the four avian species studied and 0.25 within laboratory animals, pets and humans. Next, it was also reported that although Cl and Vd might have a high intra- and interspecies variability, the T_1/2el_ can still remain stable [33]. In this respect, the Cl and Vd had a low to moderate correlation in the present study, namely R² of 0.08 and 0.79, respectively (data not shown). Despite the evidence that active renal secretion occurs in humans [35, 36], CPT is mainly eliminated unchanged by the kidneys and therefore the T_1/2el_ is primarily determined by the glomerular filtration rate (GFR). In human patients, the required dose is calculated by following formula [37]: dose (mg) = target AUC x (GFR + 25). In normally hydrated birds, the whole kidney GFR (mL/h) can be scaled to the BW (g): *Y = 1*.*24*.*W* ^*0*.*69*^ [38]. As the exponent for scaling of GFR and T_1/2el_ is different, 0.69 and 0.30, respectively, a similar formula will probably not be accurate for dose determination in bird species. These differences can be explained by species dependent differences in elimination of CPT. In rodents for instance, less than 10% of CPT is eliminated through non-renal processes (bile or irreversible binding to plasma proteins) [22], whereas in humans this is ± 30% [37]. As described above, active renal secretion occurs in humans whilst in rodents CPT is renally excreted by glomerular filtration alone [22]. Next, as only the free CPT can be eliminated, species dependent differences in plasma protein binding can further explain the lack of correlation of GFR or clearance and the BW of the species. Indeed, 4 and 24 h after administration of CPT to humans, only 24% and 40% of the CPT is bound to plasma proteins [29, 39], respectively, while in rats 65% of CPT is bound after 4 h [22].

The demonstrated correlation between T_1/2el_ and BW is a first step towards dose optimization of CPT in avian species. An appropriate dosage regimen can be calculated based on PK elimination data and the target CPT concentration for a certain type of tumour [37]. Although CPT has been shown to be successful in different cases of avian oncology, e.g. pancreatic duct adenocarcinoma [10], Sertoli cell tumour [11], and renal adenocarcinoma [12], measurements of CPT target concentration are lacking. The CPT dose in this study was therefore adjusted from the dose commonly used in chemotherapy in dogs [13]. Furthermore, dose optimization should also include data about the maximum tolerated dose. No clinical signs of CPT toxicity were observed in this trial, however blood counts or biochemistry were not evaluated since only limited amount of blood could be collected and the total volume was needed for CPT analysis. In contrast, Filippich et al. [13] observed anorexia and vomiting in cockatoos subsequently to CPT administration at a dose of 5 mg/kg BW. However, myelosuppression, which is the dose limiting side effect, has only been described in avian cases were CPT was administered at multiple doses of 11.25–15 mg/kg BW [11, 40].

## Conclusions

As a conclusion it can be stated that a sensitive and specific LC-MS/MS method was developed for quantification of free CPT in avian plasma and that, based on the PK study performed in parakeets, pigeons, ducks and chickens, the T_1/2el_ of CPT seems to be the most robust parameter for allometric scaling of CPT in avian species.

## Supporting Information

S1 Table Results of the evaluation of linearity (goodness-of-fit (g) and correlation coefficient (r)) and limit of quantification (LOQ) for quantification of free carboplatin in plasma of chickens, ducks, pigeons and parakeets. (PDF)

S2 Table Results of the within-run and between-run accuracy and precision for quantification of free carboplatin in plasma of chickens, ducks, pigeons and parakeets. (PDF)
