# Supplementary material for: Comparative Pharmacokinetics and Allometric Scaling of Carboplatin in Different Avian Species
Source: PLoS One. 2015 Jul 29;10(7):e0134177. doi: 10.1371/journal.pone.0134177 (PMC4519271; doi:10.1371/journal.pone.0134177)
Supplement: S1 Table — (PDF) [file pone.0134177.s001.pdf]

S1 Table. Results of the evaluation of linearity (goodness-of-fit (g) and correlation coefficient (r)) and limit of quantification (LOQ) for quantification of free carboplatin in plasma of chickens, ducks, pigeons and parakeets

Avian species	Calibration Range (ng/mL)	g (%)	r	LOQ (ng/mL)
Chicken	20 – 10000	4.07	0.9998	20
	500 – 50000	3.71	0.9999	
Duck	20 – 10000	3.92	0.9999	20
	500 – 50000	3.48	0.9998	
Pigeon	50 – 10000	6.10	0.9999	50
	500 – 50000	3.43	0.9997	
Parakeet	100 – 10000	5.11	0.9995	100
	200 – 50000	7.29	0.9985	

Acceptance criteria: g ≤ 10 %, r ≥ 0.99
