# Supplementary material for: Comparative Pharmacokinetics and Allometric Scaling of Carboplatin in Different Avian Species
Source: PLoS One. 2015 Jul 29;10(7):e0134177. doi: 10.1371/journal.pone.0134177 (PMC4519271; doi:10.1371/journal.pone.0134177)
Supplement: S2 Table — (PDF) [file pone.0134177.s002.pdf]

S2 Table. Results of the within-run and between-run accuracy and precision for quantification of free carboplatin in plasma of chickens, ducks, pigeons and parakeets

Avian species	Theoretical concentration (ng/mL)	Mean concentration ± SD (ng/mL)	Precision RSD (%)	Accuracy (%)
Chicken	20[a]	17.3 ± 1.33	7.7	-13.7
	50[a]	44.3 ± 1.32	3.0	-11.4
	50[b]	46.1 ± 4.07	8.8	-7.8
	500[a]	490.8 ± 5.13	1.0	-1.8
	500[b]	459.7 ± 33.26	17.2	-8.1
	5000[a]	4504.1 ± 168.95	3.8	-9.9
	5000[b]	4380.1 ± 236.55	5.4	-12.4
	20000[a]	17088.9 ± 442.97	2.6	-14.6
Duck	20[a]	19.5 ± 1.408	7.2	-2.7
	50[a]	48.4 ± 3.15	6.5	-3.2
	50[b]	45.4 ± 4.16	9.2	-9.1
	500[a]	467.9 ± 14.52	3.1	-6.4
	500[b]	484.0 ± 58.39	12.1	-3.2
	5000[a]	4674.6 ± 79.34	1.7	-6.5
	5000[b]	4600.7 ± 566.72	12.3	-8.0
	50000[a]	48432.1 ± 2486.40	5.1	-3.9
Pigeon	50[a]	47.1 ± 3.16	6.7	-5.9
	50[b]	47.8 ± 4.72	9.9	-4.4
	500[a]	428.2 ± 8.20	1.9	-14.4
	500[b]	467.2 ± 30.99	6.6	-6.6
	5000[a]	4290.3 ± 185.93	4.3	-14.2
	5000[b]	4671.4 ± 402.71	8.6	-6.6
	20000[a]	19722.7 ± 1936.54	9.8	-1.4
Parakeet	100[a]	94.4 ± 5.48	5.8	-5.6
	500[a]	479.6 ± 14.64	3.1	-4.1
	500[b]	478.9 ± 20.12	4.2	-4.2
	5000[a]	5043.6 ± 80.98	1.6	0.9
	5000[b]	5274.1 ± 202.51	3.8	5.5
	20000[a]	20427.4 ± 413.62	2.0	2.1

[a] Within-run accuracy and precision (n=6)
[b] Between-run accuracy and precision (n=6); SD: standard deviation; RSD: relative standard deviation;
Acceptance criteria: accuracy: -20% to +10%, within-run precision (RSD_{max}): 20 ng/mL: 19.2%, 50 ng/mL: 16.7%, 500 ng/mL: 11.8%, 5000 ng/mL: 8.4%, 20000 ng/mL: 6.8%, 50000 ng/mL: 5.9%; between-run precision: 50 ng/mL: 25.1%, 500 ng/mL: 17.8%, 1000 ng/mL: 16.0%, 5000 ng/g: 12.6%
